# Reducing Dietary Polyunsaturated to Saturated Fatty Acids Ratio Improves Lipid and Glucose Metabolism in Obese Zucker Rats

**DOI:** 10.3390/nu15224761

**Published:** 2023-11-13

**Authors:** Gianfranca Carta, Elisabetta Murru, Giovanna Trinchese, Gina Cavaliere, Claudia Manca, Maria Pina Mollica, Sebastiano Banni

**Affiliations:** 1 Department of Biomedical Sciences, University of Cagliari, 09042 Monserrato, Italy; m.elisabetta.murru@gmail.com (E.M.); claumanca@unica.it (C.M.); banni@unica.it (S.B.); 2 Department of Biology, University of Naples Federico II, 80126 Naples, Italy; giovanna.trinchese@unina.it (G.T.); mariapia.mollica@unina.it (M.P.M.); 3 Department of Pharmaceutical Sciences, University of Perugia, 06126 Perugia, Italy; gina.cavaliere@unipg.it

**Keywords:** dietary fat, fatty acid metabolism, obesity, insulin resistance, mitochondria, *N*-oleoylethanolamine (OEA)

## Abstract

We investigated the influence of varying dietary polyunsaturated fatty acid (PUFA)/saturated fatty acids (SFA) ratios on insulin resistance (IR), fatty acid metabolism, *N*-acylethanolamine (NAE) bioactive metabolite levels, and mitochondrial function in lean and obese Zucker rats in a model designed to study obesity and IR from overnutrition. We provided diets with 7% fat (*w*/*w*), with either a low PUFA/SFA ratio of 0.48, predominantly comprising palmitic acid (PA), (diet-PA), or the standard AIN-93G diet with a high PUFA/SFA ratio of 3.66 (control, diet-C) over eight weeks. In obese rats on diet-PA versus diet-C, there were reductions in plasma triglycerides, cholesterol, glucose, insulin concentrations and improved muscle mitochondrial function, inflammatory markers and increased muscle *N*-oleoylethanolamine (OEA), a bioactive lipid that modulates lipid metabolism and metabolic flexibility. Elevated palmitic acid levels were found exclusively in obese rats, regardless of their diet, implying an endogenous production through de novo lipogenesis rather than from a dietary origin. In conclusion, a reduced dietary PUFA/SFA ratio positively influenced glucose and lipid metabolism without affecting long-term PA tissue concentrations. This likely occurs due to an increase in OEA biosynthesis, improving metabolic flexibility in obese rats. Our results hint at a pivotal role for balanced dietary PA in countering the effects of overnutrition-induced obesity.

## 1. Introduction

Numerous epidemiological studies have indicated a correlation between elevated dietary fat intake and an increased body mass index (BMI) in humans [1]. The standard American diet derives approximately 35–40% of its energy from fat [2], which is often considered “unhealthy” as it surpasses the 30% of total energy from fat [3]. Intriguingly, a rise in obesity prevalence in the United States has been linked with a decrease in fat and calorie consumption, a phenomenon referred to as the “American paradox” [4]. A recent systematic review analyzing dietary trends in relation to the onset of non-communicable diseases identified an inverse relationship between animal fat intake and these conditions [5]. However, in scientific research, diet-induced obesity models (DIO) are frequently employed to mimic the Western dietary pattern, even though the impact of a closely formulated Western diet for rodents, taking into account macro- and micronutrient components, showed quite different effects on weight gain and biomarkers of metabolic function in mice compared to a 45% fat DIO [6]. DIO models often contain a notably high fat content, accounting for roughly 59% of energy from fat [7,8,9,10,11], which is over 3.5 times the suggested 16.7% energy from fat for growing laboratory rats [12]. Transposing these values to human equivalents, tripling dietary fat consumption would substantially surpass the tolerable fat intake limits for humans. Genetic overnutrition models, such as the *fa*/*fa* (leptin receptor) Zucker rats or the *db*/*db* and *ob*/*ob* (leptin) mice, present a more comparable representation of diet-induced human obesity conditions [13,14,15]. Furthermore, these models enable a more accurate assessment of the potential impacts of varying dietary fatty acid (FA) profiles on obese-related dysmetabolic conditions.

Different dietary FA may play distinct roles in human obesity, particularly in the regulation of inflammatory signals and insulin resistance (IR). These differences may be related to variations in oxidation rates among FA, with long-chain saturated FA (SFA) being the least oxidized [16]. Moreover, the bioactive metabolites derived from FA have the capacity to modulate lipid and energy metabolism by affecting peroxisome proliferator-activated receptor (PPAR)-α and endocannabinoid (EC) systems [17,18,19]. These differences in FA impacts may partially explain variations in weight gain and the impaired lipid and energy metabolism observed in animals fed different types of dietary fat [2,20].

In vitro studies have shown that chronic treatment with palmitic acid (16:0, PA) impairs insulin secretion and promotes the formation of intracellular cholesterol, stearic acid (18:0), C16:0 dihydroceramide, and C24:1 sphingomyelin, leading to the decreased survival of pancreatic β-cells [21]. Higher concentration of 16:0 may induce the expression of FA elongase 6 (Elovl6) [22] and stearoyl-CoA desaturase (Δ-9-desaturase, SCD). The first enzyme converts 16:0 into 18:0, and SCD converts both to the respective monounsaturated (MUFA) palmitoleic acid (16:1n7) and oleic acid (18:1n9). High dietary intake of SFA has been associated with an increase in endogenous cholesterol synthesis and plasma lipoprotein cholesterol levels, potentially leading to lipotoxicity, cellular dysfunction, and the development of metabolic syndrome [23]. Nonetheless, some randomized controlled trials failed to show an association between a reduction in SFA intake and lower total mortality or cardiovascular disease mortality [24,25]. The balance between dietary SFA/polyunsaturated fatty acids (PUFA), may well explain these contrasting results because of the importance of the correct amount of PUFA responsible for regulating serum cholesterol [26,27]. In fact, the role of dietary SFA seems to be closely dependent on their ratio with PUFA, and high levels of SFA, when accompanied by high levels of PUFA, do not appear to be associated with lipemic alterations [28,29]. Notably, a PUFA/SFA ratio of 3.75 and a MUFA/SFA ratio of 1.5 have been recommended for growth and maintenance formulations in rodent diets [30], although a recent proposal suggests a revision of the FA composition [30,31].

Dietary FA intake can modify the composition of membrane phospholipid FA and their metabolites and lipid bioactive derivatives, such as EC and *N*-acylethanolamines (NAE), which are involved in the homeostatic control of energy systems and lipid metabolism [17,18,19]. Among the SFA, PA is the most implicated FA, with crucial physiological roles in several biological functions that are often overlooked. These seem to be closely related to the regulation of its tissue concentration, dependent on the balance of its endogenous biosynthesis and dietary intake [32,33]. This balance preserves the chemical–physical properties of membrane phospholipid, which can be influenced by the length and desaturation of the FA chain [34]. A change in the tissue concentrations and metabolism of 16:0, along with its metabolites, 16:1 and 18:1, can affect various metabolic aspects of obesity and related disorders mediated by bioactive metabolites derived from membrane FA and, as such, influenced by dietary FA. However, it is often overlooked that increased tissue concentration of 16:0 and its metabolites in the form of 16:1 and 18:1 is primarily due to its biosynthesis through de novo lipogenesis, mainly from glucose in the liver and adipose tissue, rather than having a dietary origin [32,33].

Notably, most of the experimental animal studies aimed at evaluating the potential detrimental effects of 16:0 have been conducted using HFD, introducing an additional variable that does not translate directly to humans. Based on these premises, our study aimed to investigate the impact of a physiologically balanced dietary fat intake [7] enriched in 16:0, but relatively low in linoleic acid (18:2n6) and α-linolenic acid (18:3n3), on IR, FA deposition, and metabolism in lean and obese Zucker rats. This dietary intake provides sufficient FA to prevent essential FA deficiency, with a PUFA/SFA ratio of 0.5, which is similar to the ratio observed in the Italian nutrient survey [35] and typical of the Mediterranean diet. We compared this diet to the standard AIN-93G diet, which has a PUFA/SFA ratio of approximately 4:1.

## 2. Materials and Methods

### 2.1. Experimental Diets

The two diets used, manufactured at Charles Rivers Laboratories Italia srl (Via Indipendenza, Italy), were based on the AIN-93G formulation [12,30]. Both diets contained 7% fat (*w*/*w*), soybean oil used in the control diet with high PUFA/SFA ratio (diet-C) was substituted with experimental fat in the 16:0-enriched diet with a low PUFA/SFA ratio (diet-PA), thus differing only in terms of FA composition. In diet-PA, the amount of SFA was 2.5 times that of diet-C, mostly due to the 16:0 content (1.9% in diet-PA and 0.8% in diet-C), and the PUFA/SFA ratio was 3.66 in diet-C and 0.48 in diet-PA. For the complete diet FA composition, see Table 1.

### 2.2. Animals and Experimental Procedure

Twenty-four male Zucker rats (Harlan Laboratories, Indianapolis, IN, USA), 12 obese (Ob) and 12 lean (Lean), four weeks of age with an initial weight of 172 g ± 8 and 152 g ± 4, respectively, were housed in groups (*n* = 6) for one week, before being randomly assigned to the two experimental diets. Rats were kept at a constant temperature of 22 ± 2 °C and 60% relative humidity, on a 12-h light/dark cycle, with food and water available ad libitum. Following the acclimation period, two groups of 6 obese and 6 lean rats received ad libitum the diet-C (Ob-C and Lean-C, respectively), and two other groups, 6 obese and 6 lean rats, received ad libitum diet-PA (Ob-PA and Lean-PA, respectively) for 8 weeks. Animals weight, body length from tip of nose to anus, and tail length were recorded weekly across the study. Food intake was recorded every 2 days. After a 12 h fast, rats were treated with Fentanyl (100 µg/kg of body weight) and euthanized by decapitation. Adipose tissues, liver, muscle, and plasma were isolated and stored at −80°C for lipid analyses; aliquots of liver and muscle were immediately processed for mitochondrial activity. Plasma was separated from blood samples by centrifugation at 2000× *g* for 15 min at room temperature and processed for lipidomic, glucose, and liver function parameters analyses. The study was conducted in accordance with the Declaration of Helsinki, and the protocol was approved by the Animal Research Ethics Committee of the University of Cagliari, Italy (authorization n.733/2018-PR).

### 2.3. Lipid Analyses

#### 2.3.1. Reagents

The acetonitrile, methanol, chloroform, n-hexane, ethanol, acetic acid, FA standards, all HPLC/MS grade, deferoxamine mesylate, ascorbic acid, potassium hydroxide, and hydrochloric acid were purchased from Merck KGaA (Merck KGaA, Darmstadt, Germany). FA standards, including 18:2-n6, arachidonic acid (20:4n6), docosatetraenoic acid (22:4n6), 18:3n3, eicosapentaenoic acid (20:5n3), docosahexaenoic acid (22:6n3), 16:0, 16:1n7, 18:0, and 18:1n9, were obtained from Merck KGaA (Merck KGaA, Darmstadt, Germany). Internal deuterated standards for EC and NAE quantification by isotope dilution, the 2-arachidonoyl-glycerol [2H]_5_ 2-AG, *N*-arachidonoylethanolamine [2H]_8_ AEA, *N*-oleoylethanolamine [2H]_2_ OEA, and *N*-palmitoylethanolamine [2H]_4_ PEA, were purchased from Cayman Europe (Tallinn, Estonia).

#### 2.3.2. FA Analysis

Total lipids were extracted from tissues and plasma samples according to the method of Folch [36]. Total lipid quantification was performed by colorimetric determination at 600 nm, following the procedure outlined by Chiang [37]. Aliquots of the lipid fraction were mildly saponified in order to obtain FFA [38] for the high performance liquid chromatograph (HPLC) and gas chromatography (GC) analysis. The separation and identification of unsaturated FA (UFA) were carried out using an Agilent 1100 HPLC System (Agilent, Palo Alto, CA, USA) equipped with a diode array detector, as previously reported [38]. SFA were measured as FA methyl esters (FAME), through the methylation of FFA obtained via mild saponification, by a GC (Agilent, Model 6890, Palo Alto, CA, USA) equipped with a flame ionization detector (FID) [38]. The n3 highly unsaturated FA (n3HUFA) score was calculated as the percentage of the sum of n-3 FA with 20 or more carbon atoms and three or more double bonds, divided by the sum of total FA with 20 or more carbon atoms and more than three double bonds [39]:

n-3 HUFA score = (20:5n3 + 22:6n3 + 22:5n3)/(20:5n3 + 22:6n3 + 22:5n-3 + 20:3n6 + 20:4n6 + 22:4n6 + 22:5n6 + 20:3n9) × 100

#### 2.3.3. *N*-Acylethanolamines (NAE) and Endocannabinoids (EC) Analysis

Deuterated NAE and EC were added as internal standards to the samples before extraction for quantification by isotope dilution, aliquots of the lipid fraction were used for their quantification. NAE and EC quantification was carried out by an Agilent 1100 HPLC system (Agilent, Palo Alto, CA, USA) equipped with a mass spectrometry (MS) Agilent Technologies QQQ triple quadrupole 6420 with electrospray ionization (ESI) source (Agilent Technologies, Singapore), using a positive mode (ESI+), as described in [40].

### 2.4. Plasma and Hepatic Parameters

Plasma concentrations of triglycerides and cholesterol, alanine aminotransferase (ALT) and aspartate aminotransferase (AST) were measured by a colorimetric enzymatic method using commercial kits (SGM Italia, Rome, Italy and Randox Laboratories Ltd., Crumlin, UK). Blood glucose levels were determined using a rat-calibrated glucose monitor (BRIO, Ascensia, New York, NY, USA), and the plasma insulin levels were determined by ELISA (Mercodia rat insulin; Mercodia, Uppsala, Sweden). Basal fasting values of plasma glucose and insulin were used to calculate the homoeostatic model assessment (HOMA) index as [Glucose (mg/dL) × Insulin (mU/L)]/405 [41]. Specific ELISA kits were used to determine plasma levels of tumor necrosis factor-alpha (TNF-α), interleukin-1β (IL-1β) and monocyte chemoattractant protein (MCP-1) (Thermo Scientific, Rockford, Illinois, IL, USA; Biovendor R and D, Brno, Czech Republic).

### 2.5. Mitochondria Isolation and Function

Liver and limb leg muscle aliquots were finely minced and washed in a medium containing 100 mM KCl, 50 mM Tris-HCl, pH 7.5, 5 mM MgCl_2_, 1 mM EDTA, 5 mM EGTA, and 0.1% (*w*/*v*) FA-free bovine serum albumin (BSA). Tissue fragments were homogenized with the above medium (1:8, *w*/*v*) in a Potter Elvehjem homogenizer (Heidolph, Kelheim, Germany) set at 500 rpm (4 strokes = min) and filtered through sterile gauze. The hepatic homogenate was then centrifuged at 1000× *g* for 10 min, and the resulting supernatant was again centrifuged at 3000× *g* for 10 min. The mitochondrial pellet was washed twice and finally resuspended in a medium containing 80 mM LiCl, 50 mM HEPES, 5 mM Tris-PO4, 1 mM EGTA and 0.1% (*w*/*v*) FA-free BSA, pH 7.0 [42]. The muscle homogenate was centrifuged (3000× *g*, 10 min, 4 °C). The resulting supernatant was discarded, and the pellet was resuspended and centrifuged at 500× *g* for 10 min. The supernatant was centrifuged (3000× *g*, 10 min, 4 °C) and the pellet, containing the mitochondrial fraction, was washed once and resuspended in a suspension medium [43]. The protein content of the mitochondrial suspension was determined by the method of Hartree [44], using BSA as the protein standard.

Oxygen consumption in isolated mitochondria was measured with high-resolution respirometry Hansatech oxygraph (Yellow Spring Instruments, Yellow Springs, OH, USA) at the temperature of 30 °C. Isolated mitochondria were incubated in a medium (pH 7.0) containing 80 mM KCl, 50 mM HEPES, 5 mM KH_2_PO_4_, 1 mM EGTA and 0.1% (*w*/*v*) FA-free BSA to oxidize their endogenous substrates for a few minutes. Substrates were then added at the following concentrations: 10 mM succinate plus 3.75 mM rotenone; 40 µM palmitoyl-L-carnitine plus 2.5 mM malate. State 4 oxygen consumption was obtained in the absence of ADP, and State 3 oxygen consumption was measured in the presence of 0.3 mM ADP. The respiratory control ratio (RCR) was calculated as the ratio between states 3 and 4, according to Estabrook (1967) [45]. In addition, in control experiments, we assured the quality of our mitochondrial preparation by checking that contamination of mitochondria by other ATPase-containing membranes was lower than 10%, and addition of cytochrome c (3 nmol/mg protein) only enhanced the state 3 respiratory rate by approximately 10% [29,46].

#### Oxidative Stress

The rate of mitochondrial hydrogen peroxide (H_2_O_2_) release was assayed by following the linear increase in fluorescence (excitation 312, emission 420 nm) due to the oxidation of homovanillic acid in the presence of horseradish peroxidase [46]. Mitochondrial superoxide dismutase (SOD) specific activity was measured in a medium containing 0.1 mM EDTA, 2 mM KCN, 50 mM KH_2_PO_4_, pH 7.8, 20 mM cytochrome c, 5 mM xanthine, and 0.01 U of xanthine oxidase. The SOD activity was monitored spectrophotometrically (550 nm) at 25 °C, by tracking the decrease in the reduction rate of cytochrome c by superoxide radicals, generated by the xanthine–xanthine oxidase system. One unit of SOD activity is defined as the concentration of the enzyme that inhibits cytochrome c reduction by 50% in the presence of xanthine + xanthine oxidase [47].

### 2.6. Statistical Analysis

Quantitative data are presented as mean ± SEM. Statistical significance among groups was assessed by the one-way ANOVA test followed by Tukey’s correction for multiple comparisons, and statistical significances were indicated as follows: * $, £ *p* ≤0.05, ** $$, ££ *p* ≤ 0.01, *** $$$ £££ *p* ≤ 0.001. In tables * indicate significant differences compared to Lean-C; $ compared to Lean-PA-matched controls for diet; £ compared to Ob-C-matched controls for obesity condition. Correlation studies were performed using the Pearson correlation coefficient (two-tailed). All statistical analyses were conducted using GraphPad Prism 8.0.1 (GraphPad Software Inc., La Jolla, CA, USA).

## 3. Results

### 3.1. Body Composition, Food Intake, and Tissues Total Lipids

**Figure 1:**
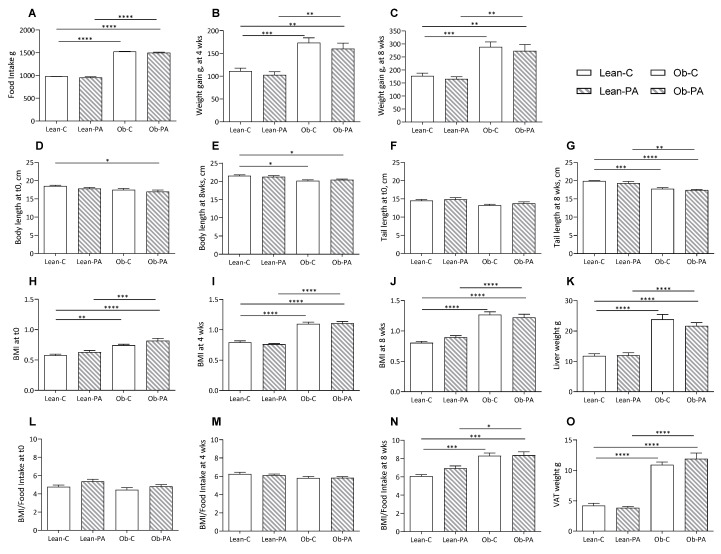
Food intake and growth parameters of lean rats fed diet-C (Lean-C) or diet-PA (Lean-PA), and obese rats fed diet-C (Ob-C) or diet-PA (Ob-PA). Food intake (**A**); weight gain at 4 weeks (**B**); and at 8 weeks (**C**); body length at t0 (**D**), and at 8 weeks (**E**); tail length at t0 (**F**), and at 8 weeks (**G**); BMI at t0 (**H**), at 4 weeks (**I**); and at 8 weeks (**J**); liver weight (**K**); BMI/Food Intake at t0 (**L**), at 4 weeks (**M**), and at 8 weeks (**N**); and visceral adipose tissue (VAT) weight (**O**). Control diet (diet-C); 16:0-enriched diet (diet-PA). Error bars represent SEM (*n* = 6) * *p* < 0.05; ** *p* < 0.01; *** *p* < 0.001; **** *p* < 0.0001.

Our results showed higher food intake after eight weeks of dietary treatment in obese rats compared to lean rats (Figure 1A), and accordingly, weight gain at four and eight weeks was significantly higher in obese compared to lean rats, irrespective of the diet (Figure 1B,C). On the contrary, body, nose–anal, and tail lengths at eight weeks were slightly reduced in obese rats regardless of the diets compared to lean rats; the body length, but not the tail length, was lower in obese rats fed diet-PA already at t0 (Figure 1D–G). BMI was higher in obese rats already at the start of the diet treatment (29%) and differently, even though not significantly, in Ob-C and Ob-PA, respectively, by 38–45% after four weeks, and by 57–36% at eight weeks compared to the lean counterparts fed the same diet (Figure 1H–J). The BMI/Food Intake ratio, that we may consider an index of the accumulation of dietary energy substrate, mainly fat, showed a steep increase in obese rats only after eight weeks of dietary treatment (Figure 1L–N).

**Figure 2:**
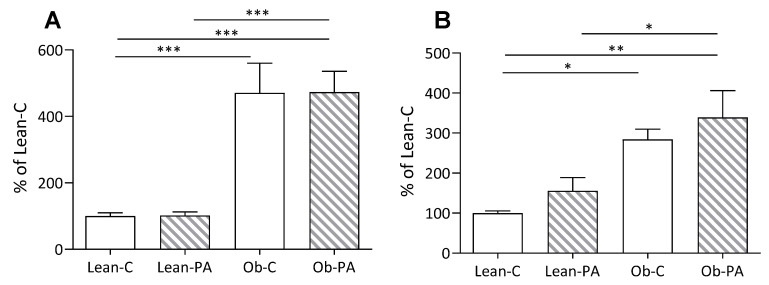
Comparison of total lipids per g of tissue: (**A**) in liver; and (**B**) in muscle, in lean rats fed diet-C (Lean-C) or diet-PA (Lean-PA), and obese rats fed diet-C (Ob-C) or diet-PA (Ob-PA). Values are expressed as % of values of Lean-C rats. Control diet (diet-C); 16:0-enriched diet (diet-PA). Error bars represent SEM (*n* = 6) * *p* < 0.05; ** *p* < 0.01; *** *p* < 0.001.

To evaluate the impact of different dietary PUFA/SFA ratios, data of each group were calculated as a percentage of Lean-C. Liver weight, and accordingly hepatic, and muscle total lipids, were increased in obese compared to lean rats, irrespective of the diet (Figure 1K and Figure 2A,B). We found an increased total amount of visceral adipose tissue (VAT) in obese rats (Figure 1O).

### 3.2. Tissue Fatty Acid Profiles

To evaluate the impact of different dietary PUFA/SFA ratios on tissue FA profile, data of each group were calculated as a percentage of Lean-C. Values of FA are shown in Table 2, Table 3, Table 4 and Table 5 for liver, AT, muscle, and plasma, respectively.

In the liver, the FA characteristic of an enhanced de novo lipogenesis, i.e., myristic acid (14:0), 16:0, 16:1n7 and 18:1n9, increased in obese rats irrespective of the diets, albeit for the latter a slight increase was also observed in Lean-PA, with a concomitant reduction in PUFA, as shown in Table 2. In contrast, obese rats showed a marked reduction in 18:0, irrespective of dietary regimen. The major change that could be ascribed to the diet and not only to obesity is on lauric acid (12:0), very abundant in diet-PA and scarcely present in diet-C, which was found to have increased in the liver of Ob-PA. A striking decrease, largely related to diet-PA, was the n3 highly unsaturated fatty acids (n3HUFA) score, although a slight reduction was also observed in obese control rats, in agreement with the lower 18:3n3 concentration in diet-PA with respect to diet-C. Nevertheless, a robust increment of MUFA and PUFAn9 was also detected in obese rats and Lean-PA.

In adipose tissue (Table 3), only 16:1n7, among the FA distinctive of de novo lipogenesis, was found to have significantly increased in obese rats, irrespective of the diets, while 16:0 also increased slightly in Lean-PA, in addition to the obese rats. Interestingly, the PA-enriched diet was able to increase 12:0, 14:0, and 18:1n9 even though the latter two FA were marginally also increased in Ob-C. Consequently, in this tissue, SFA significantly increased to the same extent in rats fed diet-PA and in Ob-C, compared to Lean-C. The 18:1n9 PUFA metabolite, 20:3n9, followed the same pattern.

As with the liver tissue, in adipose tissue, 18:3n3 and 18:2n6 were significantly decreased in relation to their concentrations in the diets, while their metabolites, long chain PUFAn3 and n6 (20:5n3, 22:6n3, 20:4n6, 22:5n6 and 22:4n6), were found to have increased in obese rats, although to a lesser extent for 22:5n6 and 22:4n6, or even reduced (20:5n3, 22:6n3, 20:4n6, and 22:4n6), in obese rats fed diet-PA, with respect to their lean counterparts. The decrease in long chain PUFAn6 and n3 in rats fed diet-PA reflected the lower concentration in the diet of their precursors, 18:3n3, 18:2n6.

In the muscle (Table 4), 16:1n7 was found to have increased, by about five times, in the obese rats irrespective of the diets, while 12:0 and 14:0 were found to have increased in the rats fed diet-PA, and 14:0 was also increased in Ob-C. 18:0 was strongly reduced in obese rats, irrespective of the diets, and in Lean-PA; an inverse trend was observed for 18:1n9. The n3HUFA score, the main PUFAs, 18:2n6, 20:4n6, and 22:6n3 were significantly decreased in relation to their precursor concentration in the diets and to the obesity condition, probably in relation to an increased accumulation of triglycerides (TG), richer in MUFA and poorer in PUFA.

In plasma, similarly to the other tissues, 16:1n7 was increased in obese rats irrespective of the diets. 18:2n6 and 18:3n3 were significantly decreased in obese rats, and diet-PA further reduced their content, reflecting their dietary concentration. The n3HUFA score and PUFA were reduced in diet-PA-fed rats (Table 5).

### 3.3. NAE and EC Tissue Profiles

**Figure 3:**
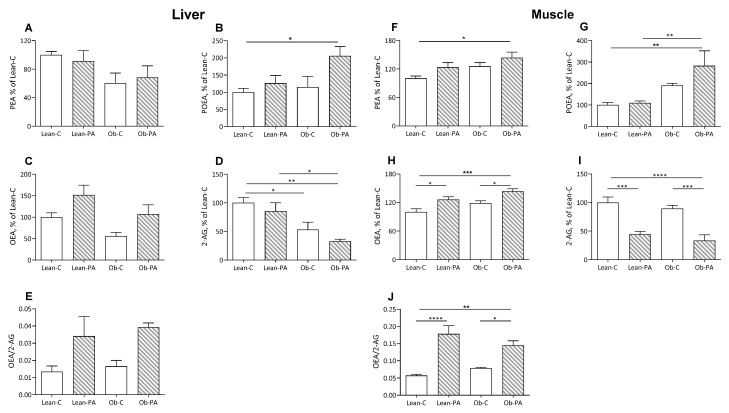
Liver and muscle levels of *N*-acylethanolamine (NAE) and 2-arachidonoylglycerol (2-AG). *N*-palmitoylethanolamine (PEA) (**A** in liver and **F** in muscle, respectively); *N*-palmitoleoylethanolamide (POEA) (**B**,**G**); *N*-oleoylethanolamine (OEA) (**C**,**H**); 2-AG (**D**,**I**); and OEA/2-AG ratio (**E**,**J**), expressed as % of values of lean rats fed diet-C (Lean-C), in lean rats fed diet-PA (Lean-PA), and obese rats fed diet-C (Ob-C) or diet-PA (Ob-PA). Control diet (diet-C); 16:0-enriched diet (diet-PA). Statistical significance among groups was assessed by one-way ANOVA followed by Tukey’s correction for multiple comparisons. Error bars represent SEM (*n* = 6) * *p* < 0.05; ** *p* < 0.01; *** *p* < 0.001; **** *p* < 0.0001.

**Figure 4:**
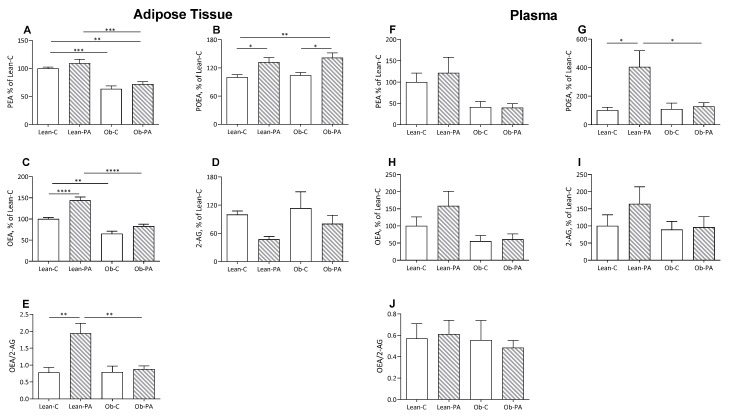
Adipose tissue and plasma levels of *N*-acylethanolamine (NAE) and 2-arachidonoylglycerol (2-AG). *N*-palmitoylethanolamine (PEA):(**A** in adipose tissue and **F** in plasma. respectively), *N*-palmitoleoylethanolamide (POEA) (**B**,**G**); *N*-oleoylethanolamine (OEA) (**C**,**H**); 2-AG (**D**,**I**); and OEA/2-AG ratio (**E**,**J**), expressed as % of values of lean rats fed diet-C (Lean-C), in lean rats fed diet-PA (Lean-PA), and obese rats fed diet-C (Ob-C) or diet-PA (Ob-PA). Control diet (diet-C); 16:0-enriched diet (diet-PA). Statistical significance among groups was assessed by one-way ANOVA followed by Tukey’s correction for multiple comparisons. Error bars represent SEM (*n* = 6) * *p* < 0.05; ** *p* < 0.01; *** *p* < 0.001; **** *p* < 0.0001.

In rats fed diet-PA, the 16:1n7 derived *N*-palmitoleoylethanolamine (POEA) was increased in the muscles and liver of obese rats, in adipose tissue in lean and obese rats, and in plasma only in the lean rats (Figure 3B,G and Figure 4B,G).

Interestingly, diet-PA increased *N*-oleoylethanolamine (OEA) levels in the muscle and, as a trend, in the liver (Figure 3C,H). In adipose tissue, OEA was increased in lean-PA, while it was reduced in obese rats irrespective of the diet; a similar but non-significant trend was observed in plasma (Figure 4C,H). In the tissues examined, *N*-palmitoylethanolamine (PEA) showed a similar pattern to OEA in adipose tissue, plasma and muscle, with the last two showing no statistically significant differences, and remained unaffected in the liver (Figure 3A,F and Figure 4A,F).

A steep reduction in 2-arachidonoylglycerol (2-AG) levels was induced by diet-PA in the muscle and, as a trend, in the adipose tissues (Figure 3I and Figure 4D), and livers of obese rats (Figure 3D).

To assess whether the different diets influence the biosynthesis of specific FA-derived ligands of PPAR-α (OEA and PEA) or EC receptor (2-AG) and, thereby, the balance between these two systems, we also evaluated the ratio between OEA/2-AG. Diet-PA increased the OEA/2-AG ratio in muscle and non-significantly also in the liver, and in adipose tissue only in Lean-PA compared to Lean-C and Ob-PA (Figure 3E,J and Figure 4E).

### 3.4. FA Correlations with NAE and EC Tissue Levels

We evaluated possible correlations between tissue concentrations of 18:2n6 and 16:0, which represent the major dissimilar FA in the two diets, and 18:1n9, highly concentrated in both diets, with their bioactive metabolites NAE and EC, ligands of PPAR-α (PEA, OEA and POEA) and EC receptors (2-AG and *N*-arachidonoylethanolamine (AEA)) (Table 6). Interestingly, 18:2n6 was found to be positively correlated with 2-AG in the muscles of both lean and obese rats and in the livers of obese rats, while, in opposition to 2-AG, OEA was negatively correlated in the muscle and adipose tissue of lean and obese rats and in the livers of obese rats. A positive correlation was found between 16:0 and OEA in lean rats AT, and with PEA in the livers of obese rats. 18:1n9 tissue concentration was also positively correlated with OEA in the adipose tissue of lean and obese rats, and negatively with POEA and AEA in the plasma of obese rats. Furthermore, there was a negative correlation with 2-AG, in lean and obese rats, in the muscle and with PEA and AEA in the livers of lean rats.

### 3.5. Plasma and Hepatic Parameters

**Figure 5:**
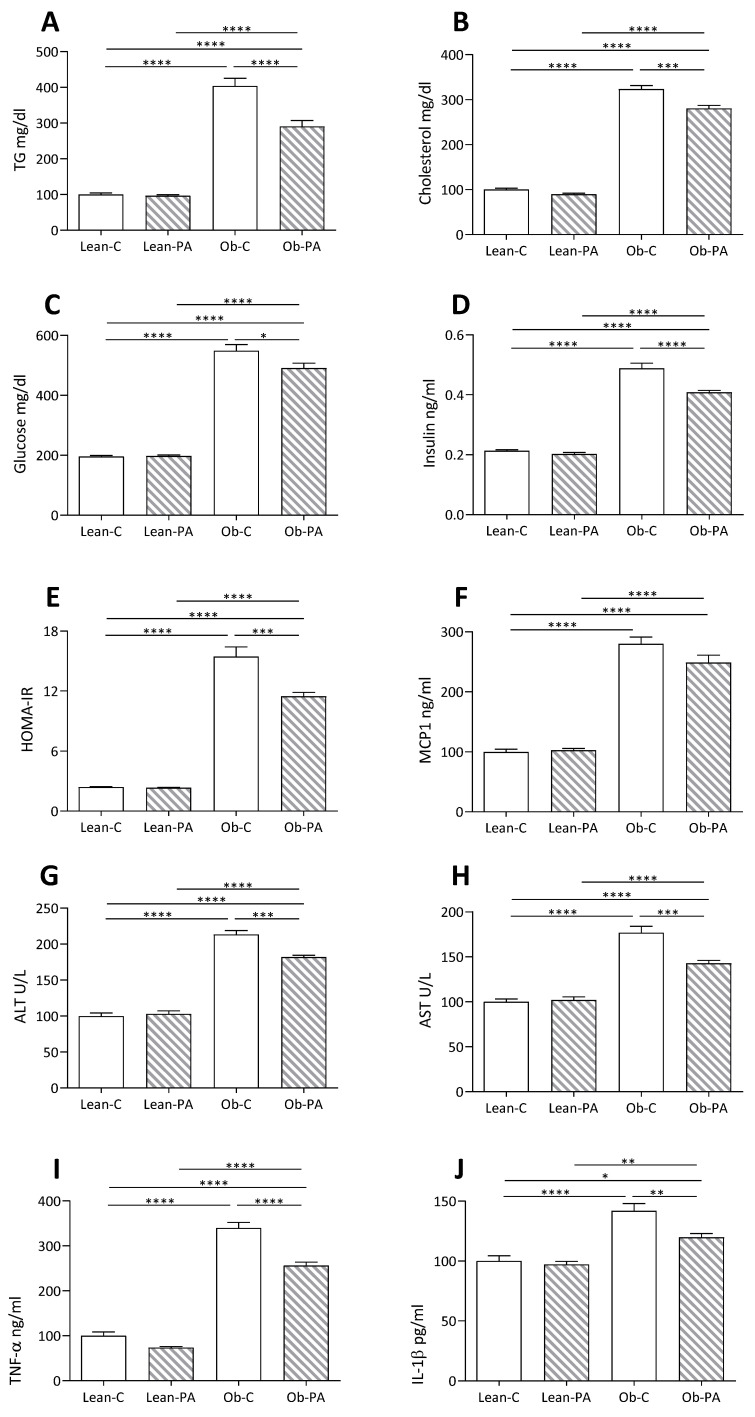
Plasma, hepatic, and inflammatory parameters: (**A**) triglycerides (TG); (**B**) cholesterol; (**C**) glucose; (**D**) insulin; (**E**) homeostasis model assessment-estimated insulin resistance (HOMA-IR); (**F**) Monocyte chemoattractant protein-1 (MCP1); (**G**) Alanine transaminase (ALT); (**H**) aspartate aminotransferase (AST); and (**I**) TNFα, (**J**) Interleukin-1 (IL-1)β, measured in lean rats fed diet-C (Lean-C) or diet-PA (Lean-PA), and obese rats fed diet-C (Ob-C) or diet-PA (Ob-PA). Control diet (diet-C); 16:0-enriched diet (diet-PA). Statistical significance among groups was assessed by one-way ANOVA followed by Tukey’s correction for multiple comparisons. Error bars represent SEM (*n* = 6) * *p* < 0.05; ** *p* < 0.01; *** *p* < 0.001; **** *p* < 0.0001.

The analysis of plasma parameters related to lipid and glucose metabolism showed a marked increase in cholesterolemia and triglyceridemia, glycemia, insulinemia and HOMA-IR (homeostasis model assessment-estimated insulin resistance) in obese rats fed both diets compared to their lean counterparts (Figure 5A–E). Interestingly, a reduction in all these parameters was observed in Ob-PA respect to Ob-C.

A similar trend was observed for liver function (ALT, AST) and inflammation (TNFα, Interleukin-1 (IL1-β) and monocyte chemoattractant protein-1 (MCP1)) parameters. However, for MCP1 the reduction observed in Ob-PA with respect to Ob-C did not reach significance (Figure 5F–J).

### 3.6. Modulation of Hepatic Mitochondrial Function and Efficiency and Oxidative Stress

**Figure 6:**
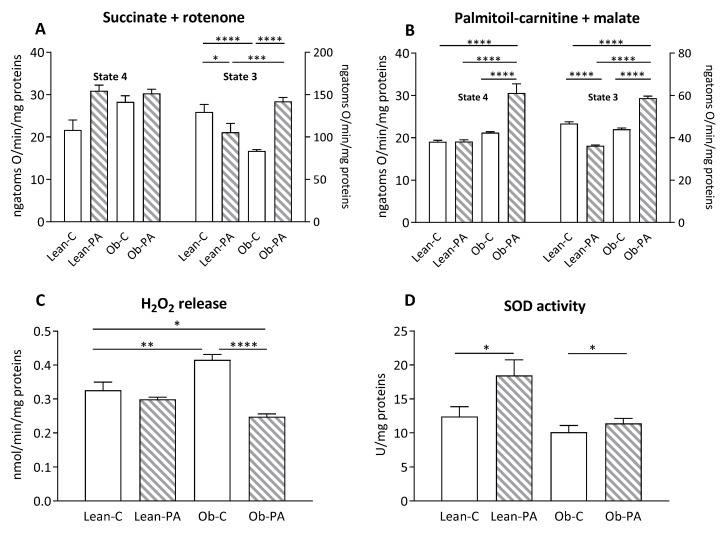
Hepatic mitochondrial respiration. The presence of succinate and rotenone (**A**); or palmitoyl-carnitine and malate (**B**) as substrates was determined in presence (state 3) or absence (state 4) of adenosine diphosphate (ADP). Hydrogen peroxide (H_2_O_2_) release (**C**); and superoxide dismutase (SOD) activity (**D**) were determined in hepatic-isolated mitochondria. Control diet (diet-C); 16:0-enriched diet (diet-PA). Statistical significance among groups was assessed by one-way ANOVA followed by Tukey’s correction for multiple comparisons. Error bars represent SEM (*n* = 6) * *p* < 0.05; ** *p* < 0.01 *** *p* < 0.001, **** *p* < 0.0001.

Mitochondrial state 3 respiratory rate, evaluated using succinate and rotenone as substrates in the presence of ADP, was decreased in lean rats fed diet-PA and in obese rats fed diet-C compared to Lean-C. Diet-PA induced a significant increase in state 3 in obese animals (Figure 6A). No variation was observed in the mitochondrial state 4 respiratory rate among all groups. When palmitoyl-carnitine and malate were used as substrates to evaluate FA oxidation in Ob-PA animals a significant increase in state 4 and state 3 mitochondrial oxygen consumption rate was observed compared to lean rats and Ob-C, whereas the lean-PA animals showed the lowest mitochondrial oxygen consumption rate in state 3 (Figure 6B). The Ob-C animals also had the highest H_2_O_2_ release compared to Lean-C and Ob-PA, while the latter group of animals showed a reduction in H_2_O_2_ release also compared to Lean-C animals (Figure 6C). The highest levels of superoxide dismutase (SOD) activity were found in diet-PA-fed rats compared to the counterparts fed diet-C (Figure 6D).

### 3.7. Modulation of Skeletal Muscle Mitochondrial Function and Efficiency and Oxidative Stress

**Figure 7:**
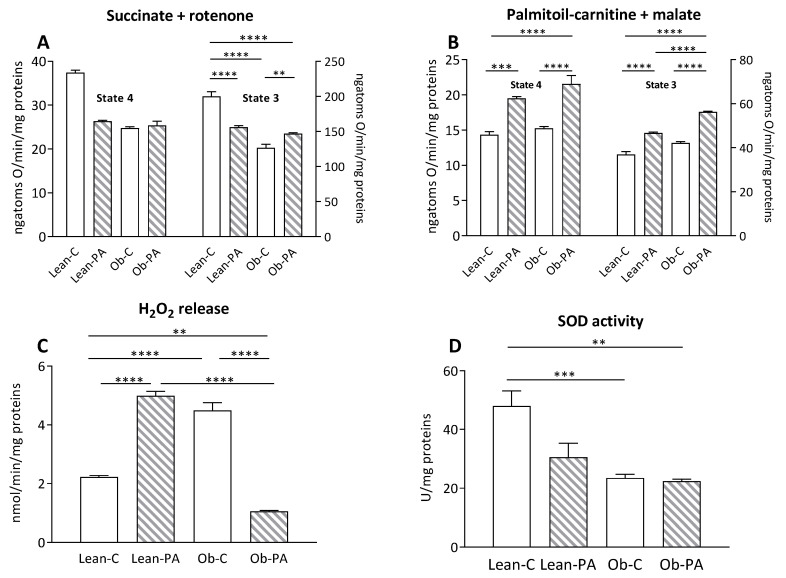
Skeletal muscle mitochondrial respiration. The presence of succinate and rotenone (**A**); or palmitoyl-carnitine and malate (**B**) as substrates was determined in the presence (state 3) or absence (state 4) of adenosine diphosphate (ADP). Hydrogen peroxide (H_2_O_2_) release (**C**); and superoxide dismutase (SOD) activity (**D**) were determined in skeletal-muscle-isolated mitochondria. Control diet (diet-C); 16:0-enriched diet (diet-PA). Statistical significance among groups was assessed by one-way ANOVA followed by Tukey’s correction for multiple comparisons. Error bars represent SEM (*n* = 6). ** *p* < 0.01 *** *p* < 0.001, **** *p* < 0.0001.

In the skeletal muscle, no variation was observed in the mitochondrial state 4 respiratory rate among all groups using succinate as a substrate (Figure 7A, left Y axis). After the addition of ADP (state 3), the mitochondrial oxygen consumption rate was reduced in obese rats and by diet-PA in lean rats, compared to Lean-C, while state 3 oxygen consumption was greater in Ob-PA compared to Ob-C (Figure 7A, right Y axis).

In addition, the animals that received diet-PA showed a significant increase in the oxidation rate of FA, in both state 4 and state 3, in comparison with respective rats fed with diet-C; furthermore, obese animals fed diet-PA had a higher state 3 oxidation rate than both groups of lean rats (Figure 7B). The dietary intake of diet-PA resulted in a significant increase in the release of H_2_O_2_ in lean animals, and, conversely, a significant reduction in this value was observed in Ob-PA compared to Ob-C (Figure 7C). SOD activity in skeletal muscle was significantly lower in obese animals, whereas the use of different diets did not result in significant changes (Figure 7D).

## 4. Discussion

Our findings demonstrate that a reduced PUFA/SFA ratio did not affect food intake or weight gain, indicating that overnutrition-induced obesity in Zucker rats is independent of dietary FA composition within a physiological dietary fat content. An intriguing study by Jeffery and colleagues, in which weanling male Lewis rats were fed an HFD (178 g fat/kg) with varying proportions of 16:0, 18:1n9, 18:2n6, and 18:3n3, revealed that food intake did not vary among animals fed different diets. However, greater weight gain and higher final body weight were observed in rats fed diets with a low PUFA/SFA ratio (0.28 and 0.81) rich in 16:0 and containing low proportions of 18:1n9 [48]. The contrast between these results and ours, which show no weight-gain differences due to dietary formulations, may be attributed to the high fat content used by Jeffery et al. compared to the normolipidic diet (despite the high 16:0 content) of our diet-PA.

In our study, there was a substantial increase in BMI in both groups of obese rats compared to lean rats, by 29% at the start of the study, and 57% in Ob-C and 36% in Ob-PA after eight weeks, with no discernible differences induced by diet composition. The BMI/Food Intake ratio, which can be considered an index of dietary energy substrate accumulation, predominantly fat, did not change after four weeks of dietary treatment. Yet, after eight weeks, lean and obese phenotypes were distinctly evident, irrespective of dietary treatment.

Body energy reserves are controlled by complex systems that regulate food intake, energy expenditure, and substrate partitioning. Therefore, an increase in the BMI/Food Intake ratio may signify excessive fat deposition leading to disrupted homeostasis in the obese phenotype, with the accumulation of ectopic fat in the liver, muscles, and plasma, and reduced metabolic flexibility, i.e., the capacity to efficiently utilize and store energy substrates based on fuel availability. This accumulation of energy substrates may be associated with an impairment related to their disposal, likely due to a metabolic inflexibility in obese rats, and may exacerbate glucose and lipid metabolism and promote inflammation. Metabolic inflexibility, which hinders the effective utilization of energy substrates for body growth, might account for the slightly shorter body and tail lengths observed in obese rats, regardless of diet.

Excessive dietary intake of SFA is usually associated with increased obesity-related hepatic inflammatory plasma markers such as ALT and AST [49,50,51], as well as cytokines, such as TNFα and IL-1β, involved in the inflammatory response. Our results confirm that obesity induced liver damage and an inflammatory status; however, these markers were significantly reduced in Ob-PA rats compared to Ob-C rats.

Therefore, our data corroborate that obesity triggers systemic inflammation, lipid and glucose metabolic impairment, IR, de novo lipogenesis, steatosis, and liver damage. However, unexpectedly, we found that diet-PA improved insulin sensitivity, reduced inflammation, and mitigated liver damage in obese rats. These findings suggest that the harmful effects previously attributed to dietary PA may be due to the extremely high fat content rather than to PA itself.

We then explored possible mechanisms through which diet-PA exerted these positive effects in obese rats. The benefits do not seem to be directly associated with tissue PA concentrations. As already evidenced by others [52], we did not observe an increase in tissue PA correlating with its dietary intake, likely due to a dilution effect, since 16:0 is, along with 18:1n9, the most abundant FA present in the body [53]. In all tissues, we found an increase in PA related to obesity irrespective of the diet, except for a minor increase (18%) in the adipose tissue of Lean-PA. This suggests that the increase in 16:0 is more likely due to increased endogenous biosynthesis related to IR in obese rats [54], rather than to its dietary intake. Chronic nutritional imbalance or pathophysiological conditions like obesity can strongly induce de novo lipogenesis in the liver and, to a lesser extent, in adipose tissue. This can lead to the overproduction of PA, resulting in an abnormal systemic inflammatory response and metabolic dysregulation, potentially causing dyslipidemia, IR, altered fat deposition, and other pathological conditions [55].

Abdominal obesity predisposes to hepatic steatosis, via both the increased free fatty acid delivery to the liver and the hyperinsulinemia-induced increase in hepatic de novo lipogenesis [56]. The sustained hepatic lipogenesis in obese rats was also evident from the increased 14:0 levels, similar to the 16:0 levels. In line with this, we observed an increase in liver weight and total hepatic lipids, indicating steatosis in obese rats, independent of dietary intake. This is likely related to obesity status, since lean rats fed diet-PA showed no further increase in hepatic lipid accumulation. While 16:0 and 16:1n7 were predominantly of endogenous origin, the 18:1n9 concentration was related to both dietary sources and endogenous biosynthesis. To maintain a stable 16:0 tissue concentration, its higher availability might induce prompt elongation and desaturation to 18:1n9 [57,58], preventing surplus accumulation. Consistently, 18:0 was significantly reduced in the livers of obese rats.

We assessed the impact of diet-PA on the tissue concentration of FA-related bioactive metabolites, such as EC and NAE, as these can modulate glucose and lipid metabolism through the PPAR-α and EC systems [17,18,19], particularly under obesity conditions.

Diet-PA led to an increase in OEA levels in muscle, liver (trending), and adipose tissue in lean rats. OEA acts as a bioactive signal for the regulation of feeding and energy homeostasis, promoting satiety [59], reducing lipogenesis [60], and possessing analgesic and anti-inflammatory properties through the activation of PPAR-α [61]. This nuclear receptor regulates appetite, food intake, energy homeostasis, lipid metabolism, and inhibits lipogenesis. Its activation may play a significant role in decreasing TG levels in plasma [62], which may promote glucose homeostasis and insulin sensitivity. Dietary PA administration increased POEA levels in adipose tissue in both lean and obese rats, and in the liver and muscles of obese rats. Like OEA, this NAE is purported to act by binding PPAR-α, promoting increased FA oxidation and a reduction in inflammation [63]. Interestingly, OEA and POEA also bind to the orphan G protein-coupled receptor 119 (GPR119), which has been shown to be able to stimulate the release of glucagon-like peptide-1 (GLP-1) from neuroendocrine cells [64], thus improving insulin action. The precursor to POEA, 16:1n7, was notably increased in obese rats, most likely deriving from de novo lipogenesis, potentially masking that from PA desaturation of dietary origin. This FA is considered a lipokine that improves insulin sensitivity [65,66,67].

Conversely, we found that different dietary concentrations of 18:2n6 substantially influenced its tissue levels and related desaturation and elongation metabolites. We thus investigated whether 18:2n6 tissue levels impact the concentrations of the EC AEA and 2-AG, both derivatives of 20:4n6, which may profoundly affect lipid and glucose metabolism. In human studies, 2-AG has been shown to positively correlate with decreased high-density lipoprotein cholesterol, and increased TG levels and IR [68,69]. It has been demonstrated that AEA and 2-AG levels were significantly elevated by a high dietary content of 18:2n6 in a low-fat diet and were associated with greater weight gain, adipogenicity, larger adipocytes, and macrophage infiltration in adipose tissue, compared to an isocaloric low 18:2n6 diet [70]. In our study, 18:2n6 liver and muscle concentrations were positively correlated with 2-AG and AEA, and negatively correlated with PPAR-α ligands (OEA, PEA and POEA), suggesting that tissue 18:2n6 modulates bioactive metabolites favoring the predominance of the EC biosynthesis over PPAR-α ligands. Consequently, a reduced content of 18:2n6, as in diet-PA, led to a reduction in 2-AG levels compared to diet-C, confirming that a diet rich in 18:2n6 may favor an overactive EC system [70].

We analyzed the OEA/2-AG ratio to provide an indication of the balance between the PPAR-α system and the EC system (supported by 2-AG). This balance may regulate the homeostatic control of energy metabolism and body composition. This ratio was elevated in rats fed diet-PA in muscle tissue, and also trended higher in liver and adipose tissue, suggesting the dominance of PPAR-α activity.

The PA-derived lipid mediator PEA exhibited a distribution pattern in the muscle similar to that of OEA, although it did not reach statistical significance. PEA is known to play an essential role in controlling the genesis of inflammation [71] that may be exerted by acting as an agonist of the nuclear receptor PPAR-α and promoting the catabolism of proinflammatory eicosanoids by inducing peroxisomal β-oxidation [59]. PPAR-α regulates the transcription of genes involved in the peroxisomal and microsomal oxidation of FA, thereby controlling serum levels of TG and cholesterol [72,73]. Furthermore, it has been shown that OEA and PEA improve metabolic flexibility [43,74]. This led us to investigate whether the balance between the PPAR-α and the EC system influences mitochondrial function, particularly in the liver and muscle.

In obese rats on diet-PA, we observed a beneficial impact on the restoration of mitochondrial respiratory activity, as evidenced by the heightened oxygen consumption rate with the FADH-linked (succinate) substrate. Notably, diet-PA had a pronounced effect on obese animals when we examined the mitochondrial FA oxidation rate using palmitoyl-carnitine as a substrate. Moreover, this increase in hepatic mitochondrial respiratory activity in Ob-PA rats did not result in an elevation in ROS production, unlike what was found in obese animals on diet-C. In fact, diet-C-fed obese rats displayed the highest H_2_O_2_ release and no variation in SOD activity.

Further, given the established role of skeletal muscle in metabolic flexibility, due to its association with mitochondrial dysfunction and IR [75], we decided to evaluate the effects of diet-PA in modulating mitochondrial function in this tissue. Diet-PA led to an increase in mitochondrial FA oxidation rates both in lean and obese animals. This data led us to propose a metabolic shift towards the oxidation of FA in the skeletal muscle of diet-PA-fed animals.

Therefore, our data strongly suggest that the increase in muscle and liver of OEA, PEA and POEA, PPAR-α ligands, and the reduction in EC 2-AG, by enhancing the ratio between PPAR-α/EC system, might promote mitochondrial function and thereby improve glucose and lipid metabolism in dysmetabolic conditions such as obesity.

However, despite the significant results observed in circulating molecules related to IR and lipid impairment, and markers of inflammation and mitochondrial functions, we did not observe a reduction in weight gain or improvement in the altered depots of body energy substrate. We theorize that significant changes in body composition might require a longer dietary treatment, suggesting that changes in metabolic flexibility could precede possible changes in body fat deposition and distribution.

A significant improvement in glucose and lipid metabolism in obese conditions has been demonstrated to be greatly influenced by long-chain PUFAn3 20:5n3 and 22:6n3, particularly in the phospholipid form, and, more specifically, by the balance between n3/n6 [38,76]. Indeed, replacing lard, rich in SFA, with fish oil (rich in PUFAn3) in HFD can limit the development of systemic and tissue inflammation, reduce fat mass and IR associated with fat overnutrition, by modulating energy efficiency. In particular, at the skeletal muscle level, the PUFAn3-enriched diet promotes mitochondrial function and thereby metabolic flexibility [29,77].

Very low levels of 18:3n3 in diet-PA led to a systemic reduction in the n3HUFA score, which is considered a biomarker of n3 FA intake and tissue status, in all tissues in both lean and obese rats [39]. Therefore, an addition of PUFAn3 to diet-PA might further improve glucose and lipid metabolism and metabolic flexibility. In practical terms, to reach an optimal 4:1 n6/n3 ratio in diet-PA, it would be sufficient to add 0.2% of 18:3n3 in the diet. A limitation of the present study, which could be addressed in future studies, is the absence of a group fed a diet with an intermediate PUFA/SFA ratio; indeed, we exclusively investigated diets with extremely low and high values of this ratio. Future studies could also explore the impact of diets containing varying proportions of MUFA and a higher n3/n6 PUFA ratio. It is worth noting that diets rich in PUFAn6 have been shown to inhibit the formation of highly PUFAn3 [78], even though a recent comprehensive review suggests that augmenting n3, n6, or total PUFA has minimal or no effects on the prevention and treatment of type 2 diabetes mellitus [79].

## 5. Conclusions

Our study unveils novel perspectives on PA, as it shows that long-term dietary PA did not significantly influence its tissue concentration, implying potential physiological mechanisms to limit an excess of tissue PA concentrations. Moreover, in obesity, regardless of the diet, the increase in PA and its metabolites sustained by enhanced de novo lipogenesis might contribute to the detrimental metabolic effects of obesity. A lower PUFA/SFA ratio, reducing dietary 18:2n6 levels balanced by 16:0 and 18:1, can decrease EC, 2-AG, and increase PPAR-α ligands in muscle, improving metabolic flexibility, mitochondrial function, and fat and glucose metabolism, without affecting weight and growth.

Of particular interest, the ratio of PUFA/SFA in human milk is 0.4 [80], mirroring the experimental diet we scrutinized in this study. Our earlier research aligns with our present observations, indicating that the inclusion of human milk in the diet of rats enhances mitochondrial function and elevates OEA concentrations in skeletal muscle [81], consequently improving metabolic flexibility. This finding suggests that maintaining this ratio could potentially be beneficial at all stages of life.

Overall, these findings propose that balanced dietary PA may play a key role in reversing metabolic derangement caused by overnutrition-induced obesity.

## Figures and Tables

**Table 1:** Principal FA, as % of diet (*w*/*w*), in control diet with high PUFA/SFA ratio (diet-C) or in diet with low PUFA/SFA ratio (diet-PA) ^1^.

FA	Diet-C	Diet-PA	(Diet-PA)-(Diet-C)
	% of the diet		
**12:0**	0.003	0.43	+0.427
**14:0**	0.02	0.19	+0.17
**16:0**	0.84	1.91	+1.07
**18:0**	0.26	0.26	+0.00
**18:1n9**	1.78	2.88	+1.10
**18:2n6**	3.69	1.23	−2.46
**18:3n3**	0.41	0.10	−0.31
**SFA**	1.12	2.79	+1.67
**MUFA**	1.78	2.88	+1.10
**PUFA**	4.10	1.33	−2.77
**PUFA/SFA**	3.66	0.48	−3.18
**MUFA/SFA**	1.59	1.03	−0.56
**n3/n6**	0.41	0.08	−0.33
**total FA**	7.00	7.00	0.00

^1^ Diets were AIN-93G by Harlan, soybean oil in diet-C was substituted with experimental fats in diet-PA. SFA (saturated FA), n3/n6 (PUFAn3/PUFAn6).

**Table 2:** Most concentrate liver FA.

	Lean-C		Lean-PA		Ob-C		Ob-PA	
FA in Liver	Mean	SEM	Mean	SEM	Mean	SEM	Mean	SEM
**12:0**	100	16.18	161.21	28.28	103.20	16.35	297.37	48.18 *** $ ££
**14:0**	100	19.32	110.54	15.70	314.52	36.33 ***	331.37	22.69 *** $$$
**16:0**	100	6.53	108.85	4.10	162.83	10.47 ***	167.89	5.12 *** $$$
**18:0**	100	7.55	104.71	5.60	36.32	4.13 ***	42.42	2.71 *** $$$
**14:1n5**	100	21.31	87.55	23.51	215.45	35.57	271.84	40.78 ** $$
**16:1n7**	100	13.80	121.45	15.98	462.09	35.65 ***	465.15	14.86 *** $$$
**18:1n9**	100	11.23	175.04	22.19 *	299.65	16.34 ***	326.24	13.38 *** $$$
**18:3n3**	100	23.63	23.04	2.48 **	50.47	14.73	14.51	1.72 **
**20:5n3**	100	24.61	33.51	0.91	58.66	23.25	14.64	1.98 *
**22:6n3**	100	6.36	64.54	5.88 ***	23.44	5.35 ***	16.73	1.23 *** $$$
**18:2n6**	100	7.44	49.25	3.49 ***	43.06	4.95 ***	18.44	1.72 *** $$ ££
**18:3n6**	100	11.71	55.76	4.51	101.36	19.13	44.55	5.98 * £
**20:4n6**	100	5.87	98.49	7.67	25.25	4.96 ***	25.41	1.49 *** $$$
**22:4n6**	100	13.17	93.81	12.02	51.09	13.50	35.71	3.60 * $
**20:3n9**	100	41.28	139.71	18.60	39.42	7.46	64.98	6.98 $
**SFA**	100	6.58	106.36	5.62	110.77	6.46	116.32	3.75
**MUFA**	100	12.60	166.51	20.96 *	337.97	19.35 ***	361.31	10.75 *** $$$
**PUFA**	100	5.24	79.22	5.71 *	33.99	5.56 ***	24.39	1.73 *** $$$
**PUFAn3**	100	6.91	59.96	4.96 ***	27.02	6.11 ***	16.98	1.10 *** $$$
**PUFAn6**	100	5.24	82.02	6.13 *	34.96	5.34 ***	24.81	1.76 *** $$$
**PUFAn9**	100	10.79	175.87	21.30 *	293.85	15.75 ***	320.73	13.01 *** $$$
**n3HUFA score**	100	4.31	65.77	1.68 ***	82.51	3.45 **	60.39	2.52 *** £££

Values of FA are presented as means ± SEM (*n* = 6/group) expressed as % of the FA of lean rats fed diet-C (Lean-C), in lean rats fed diet-PA (Lean-PA), and obese rats fed diet-C (Ob-C) or diet-PA (Ob-PA). Statistical significance among groups was assessed by one-way ANOVA followed by Tukey’s correction for multiple comparisons, * $ £ *p* ≤ 0.05, ** $$ ££ *p* ≤ 0.01, *** $$$ £££ *p* ≤ 0.001, significant difference. * Indicate significant differences compared to Lean-C; $ indicate significant differences compared to Lean-PA-matched controls for diet; £ indicate significant differences compared to Ob-C-matched controls for obesity condition. Control diet (diet-C); 16:0-enriched diet (diet-PA); n3HUFA score (n3 Highly Unsaturated FA); MUFA (monounsaturated FA); SFA (saturated FA).

**Table 3:** Most concentrate adipose tissue (AT) FA.

	Lean-C		Lean-PA		Ob-C		Ob-PA	
FA in AT	Mean	SEM	Mean	SEM	Mean	SEM	Mean	SEM
**12:0**	100	9.50	751.58	158.74 ***	61.35	10.96	335.97	72.98 $$
**14:0**	100	4.96	157.02	2.74 ***	118.75	2.76 **	138.43	3.27 *** $$ ££
**16:0**	100	1.73	118.24	1.57 ***	126.44	2.81 ***	129.94	2.13 *** $$
**18:0**	100	3.84	82.18	4.13	92.81	9.17	80.06	6.31
**14:1n5**	100	7.38	126.4	36.99	282.3	72.82	318.97	102.03
**16:1n7**	100	5.99	141.60	18.42	253.89	38.60 **	291.07	40.62 *** $$
**18:1n9**	100	2.15	163.63	1.23 ***	116.27	1.61 ***	152.64	1.29 ***$$ £££
**18:3n3**	100	3.15	17.78	1.80 ***	54.35	1.73 ***	12.27	0.60 *** £££
**20:5n3**	100	8.15	36.89	8.93 ***	176.71	4.67 ***	51.47	7.20 *** £££
**22:6n3**	100	6.80	26.36	3.90 ***	182.64	18.40 ***	39.99	6.14 *** £££
**18:2n6**	100	2.52	33.29	1.67 ***	50.75	0.59 ***	20.02	0.68 *** $$$ £££
**18:3n6**	100	2.84	24.22	3.23 ***	72.08	5.17 ***	24.79	1.91 *** £££
**20:4n6**	100	6.23	52.06	9.14 ***	128.17	3.10 *	58.04	2.50 *** £££
**22:5n6**	100	5.78	91.49	13.55	179.19	9.53 ***	125.86	10.60 $$ £££
**22:4n6**	100	8.36	45.00	15.50 ***	163.81	11.10 ***	61.63	5.91 * £££
**20:3n9**	100	7.25	185.01	14.87 **	199.51	9.22 ***	322.49	23.94 *** $$$ £££
**SFAs**	100	2.12	123.17	0.72 ***	120.06	3.17 ***	125.53	1.52 ***
**MUFA**	100	1.94	158.39	2.77 ***	134.41	4.30 ***	170.70	3.75 *** £££
**PUFA**	100	2.43	33.36	1.68 ***	55.25	0.67 ***	21.98	0.69 *** $$$ £££
**PUFAn3**	100	3.25	18.19	1.80 ***	61.30	1.93 ***	13.95	0.78 *** £££
**PUFAn6**	100	2.44	34.06	1.72 ***	53.41	0.60 ***	21.58	0.73 *** $$$ £££
**PUFAn9**	100	2.15	163.67	1.26 ***	116.49	1.60 ***	153.09	1.28 *** $$$ £££
**n3HUFA score**	100	5.42	47.10	2.95 ***	108.96	6.76	51.96	6.47 *** £££

Values of FA are presented as means ± SEM (*n* = 6/group) expressed as % of the FA of lean rats fed diet-C (Lean-C), in lean rats fed diet-PA (Lean-PA), and obese rats fed diet-C (Ob-C) or diet-PA (Ob-PA). Statistical significance among groups was assessed by one-way ANOVA followed by Tukey’s correction for multiple comparisons, * *p* ≤ 0.05, ** $$ ££ *p* ≤ 0.01, *** $$$ £££ *p* ≤ 0.001, significant difference. * Indicate significant differences compared to Lean-C; $ indicate significant differences compared to Lean-PA-matched controls for diet; £ indicate significant differences compared to Ob-C-matched controls for obesity condition. Control diet (diet-C); 16:0-enriched diet (diet-PA); n3HUFA score (n3 Highly Unsaturated FA); MUFA (monounsaturated FA); SFA (saturated FA).

**Table 4:** Most concentrate muscle FA.

	Lean-C		Lean-PA		Ob-C		Ob-PA	
FA in Muscle	Mean	SEM	Mean	SEM	Mean	SEM	Mean	SEM
**12:0**	100	14.60	938.73	150.72 ***	108.02	11.98	639.02	28.29 *** £££
**14:0**	100	9.38	190.96	23.22 ***	160.17	9.73 *	227.50	7.27 *** £
**16:0**	100	2.76	98.80	8.03	100.41	6.72	113.71	3.08
**18:0**	100	10.21	65.67	8.23 *	38.81	4.95 ***	39.56	3.77 ***
**14:1n5**	100	45.35	83.64	34.14	81.89	10.89	53.15	14.26
**16:1n7**	100	16.55	148.51	33.59	480.03	48.16 ***	517.92	46.40 *** $$$
**18:1n9**	100	12.12	187.65	29.75 **	186.26	6.75 **	218.53	6.22 ***
**18:3n3**	100	14.62	29.71	5.25 ***	117.41	13.27	28.84	2.80 *** £££
**20:5n3**	100	16.21	51.19	11.35 *	134.08	9.55	66.17	7.20 ££
**22:6n3**	100	10.36	44.86	7.64 ***	41.18	3.67 ***	26.81	2.77 ***
**18:2n6**	100	6.89	55.94	4.61 ***	80.81	3.18 *	40.73	2.13 *** £££
**18:3n6**	100	11.08	53.51	5.93 *	131.46	12.96	57.55	1.82 * £££
**20:4n6**	100	9.67	74.06	12.34	51.89	3.43 **	43.89	3.53 ***
**22:5n6**	100	8.47	166.04	18.96 **	88.45	10.09	113.29	13.08
**22:4n6**	100	6.31	88.34	12.76	99.77	6.40	76.50	6.99
**20:3n9**	100	40.50	275.93	37.52 *	169.98	9.11	434.43	27.57 *** $
**SFA**	100	4.74	107.02	9.26	81.73	5.14	95.50	2.20
**MUFA**	100	12.52	180.92	29.86 *	234.65	12.79 ***	267.22	11.59 *** $
**PUFA**	100	4.49	64.65	7.57 ***	74.52	2.38 **	44.07	2.82 *** $ ££
**PUFAn3**	100	7.84	44.13	6.48 ***	54.86	2.52 ***	29.57	2.21 *** £
**PUFAn6**	100	5.91	65.24	6.29 ***	74.24	2.47 **	44.28	3.06 *** $ ££
**PUFAn9**	100	12.24	189.50	29.45 **	186.95	6.85 **	221.65	6.08 ***
**n3HUFA score**	100	5	65.64	2.75 ***	77.77	1.81 **	62.77	3.72 *** £

Values of FA are presented as means ± SEM (*n* = 6/group) expressed as % of the FA of lean rats fed diet-C (Lean-C), in lean rats fed diet-PA (Lean-PA), and obese rats fed diet-C (Ob-C) or diet-PA (Ob-PA). Statistical significance among groups was assessed by one-way ANOVA followed by Tukey’s correction for multiple comparisons, * $ £ *p* ≤ 0.05, ** ££ *p* ≤ 0.01, *** $$$ £££ *p* ≤0.001, significant difference. * Indicate significant differences compared to Lean-C; $ indicate significant differences compared to Lean-PA-matched controls for diet; £ indicate significant differences compared to Ob-C-matched controls for obesity condition. Control diet (diet-C); 16:0-enriched diet (diet-PA); n3HUFA score (n3 Highly Unsaturated FA); MUFA (monounsaturated FA); SFA (saturated FA).

**Table 5:** Most concentrate plasma FA.

	Lean-C		Lean-PA		Ob-C		Ob-PA	
FA In Plasma	Mean	SEM	Mean	SEM	Mean	SEM	Mean	SEM
**12:0**	100	18.78	209.46	34.44 *	38.44	4.61	105.08	3.43 $
**14:0**	100	7.14	137.55	13.40	109.90	23.35	140.18	12.29
**16:0**	100	7.78	123.45	8.61	111.39	19.95	124.35	6.88
**18:0**	100	5.74	107.32	9.12	91.96	19.56	111.69	9.52
**14:1n5**	100	27.11	54.79	13.70	33.51	6.42 *	49.57	3.60
**16:1n7**	100	10.68	162.83	21.17	381.26	57.99 **	395.88	41.91 *** $$
**18:1n9**	100	11.87	178.24	19.68	165.94	26.62	188.75	14.83 *
**18:3n3**	100	8.68	22.27	1.57 ***	59.53	13.42 *	15.26	1.95 *** ££
**20:5n3**	100	10.36	36.26	2.44	154.81	24.57	57.19	4.29 ££
**22:6n3**	100	8.39	56.26	6.59 **	84.50	9.15	60.55	7.46 *
**18:2n6**	100	6.08	46.45	2.41 ***	53.58	8.14 ***	24.21	1.71 *** $ ££
**18:3n6**	100	7.74	81.66	7.74	111.12	4.14	72.39	5.17 * £
**20:4n6**	100	12	80.11	9.06	73.88	18.99	71.51	12.83
**22:4n6**	100	14.21	84.01	10.36	162.83	36.15	106.72	10.96
**22:5n6**	100	7.94	345.81	21.10 ***	232.90	35.34 *	436.17	21.48 *** £££
**20:3n9**	100	6.14	441.52	88.73 **	270.42	88.73	710.60	47.49 *** $ £££
**SFA**	100	6.12	119.03	8.04	120.82	17.15	119.25	4.38
**MUFA**	100	7.70	149.22	18.45	337.44	50.38 **	352.24	36.99 ** $$
**PUFA**	100	5.45	67.55	5.27 *	72.67	7.55 *	57.62	5.59 **
**PUFAn3**	100	4.80	49.71	4.69 ***	87.07	5.51	53.10	4.63 *** £££
**PUFAn6**	100	5.77	65.22	5.41 *	68.09	8.00 *	51.76	5.89 ***
**PUFAn9**	100	11.72	180.56	19.41	167.23	26.64	193.36	14.62 *
**n3HUFA score**	100	2.89	62.55	0. 96 **	109.14	9.90	69.45	1.84 * ££

Values of FA are presented as means ± SEM (*n* = 6/group) expressed as % of the FA of lean rats fed diet-C (Lean-C), in lean rats fed diet-PA (Lean-PA), and obese rats fed diet-C (Ob-C) or diet-PA (Ob-PA). Statistical significance among groups was assessed by one-way ANOVA followed by Tukey’s correction for multiple comparisons, * $ £ *p* ≤ 0.05, ** $$ ££ *p* ≤ 0.01, *** £££ *p* ≤0.001, significant difference. * Indicate significant differences compared to Lean-C; $ indicate significant differences compared to Lean-PA-matched controls for diet; £ indicate significant differences compared to Ob-C-matched controls for obesity condition. Control diet (diet-C); 16:0-enriched diet (diet-PA); n3HUFA score (n3 Highly Unsaturated FA); MUFA (monounsaturated FA); SFA (saturated FA).

**Table 6:** Tissue correlations ^1^ between 16:0, 18:1n9, 18:2n6 and NAE or EC molecules in Lean or Obese Zucker rats.

		Liver				Muscle				Plasma				AT			
	NAE	Lean		Obese		Lean		Obese		Lean		Obese		Lean		Obese	
	EC	r	*p*	r	*p*	r	*p*	r	*p*	r	*p*	r	*p*	r	*p*	r	*p*
**16:0**	PEA	0.062	0.856	**0.645**	**0.024**	0.026	0.937	0.507	0.929	0.409	0.275	−0.116	0.719	−0.043	0.873	0.081	0.765
	OEA	0.121	0.724	0.396	0.202	0.339	0.282	0.461	0.131	0.598	0.089	−0.023	0.943	**0.674**	**0.004**	0.270	0.312
	POEA	0.045	0.895	0.530	0.076	0.068	0.834	0.461	0.132	0.188	0.655	−0.167	0.605	−0.043	0.873	0.081	0.765
**18:1n9**	AEA	**−0.847**	**0.001**	−0.460	0.133	0.144	0.655	0.225	0.482	0.210	0.561	**−0.644**	**0.024**	0.255	0.358	−0.362	0.169
	2-AG	−0.401	0.221	−0.351	0.290	**−0.740**	**0.006**	**−0.710**	**0.010**	−0.244	0.527	0.086	0.792	−0.256	0.338	−0.212	0.430
	PEA	**−0.768**	**0.006**	−0.431	0.162	0.222	0.489	0.086	0.790	−0.472	0.200	−0.491	0.105	0.058	0.831	−0.125	0.646
	OEA	−0.086	0.801	−0.172	0.594	0.236	0.460	0.383	0.219	−0.117	0.765	−0.423	0.171	**0.799**	**0.0002**	**0.507**	**0.045**
	POEA	0.296	0.376	−0.183	0.570	−0.023	0.944	0.179	0.578	0.264	0.528	**−0.752**	**0.005**	0.058	0.831	−0.125	0.646
**18:2n6**	AEA	**0.650**	**0.031**	0.090	0.793	−0.457	0.135	−0.479	0.115	−0.289	0.418	−0.419	0.176	−0.198	0.480	0.250	0.350
	2-AG	0.169	0.619	**0.672**	**0.024**	**0.624**	**0.030**	**0.898**	**0.0001**	−0.255	0.477	0.003	0.992	0.251	0.349	0.199	0.460
	PEA	0.159	0.640	−0.312	0.324	−0.741	0.006	−0.352	0.263	−0.043	0.913	−0.271	0.394	−0.011	0.968	0.097	0.721
	OEA	−0.424	0.194	**−0.585**	**0.046**	**−0.803**	**0.002**	**−0.719**	**0.008**	−0.423	0.257	−0.333	0.290	**−0.739**	**0.001**	**−0.544**	**0.029**
	POEA	−0.211	0.533	**−0.603**	**0.038**	−0.243	0.447	−0.339	0.281	−0.617	0.103	−0.512	0.089	−0.011	0.968	0.097	0.721

^1^ Pearson correlation coefficients. *p* (Two tailed) bold values indicate significance *p* ≤ 0.05. *N*-acylethanolamine (NAE), *N*-oleoylethanolamine (OEA), *N*-palmitoleoylethanolamine (POEA), *N*-palmitoylethanolamine (PEA), endocannabinoids (EC) *N*-arachidonoylethanolamine (AEA), 2-arachidonoylglycerol (2-AG).

## Data Availability

The data presented in this study are available on request from the corresponding author: Gianfranca Carta Department of Biomedical Sciences, University of Cagliari, 09042 Monserrato, Italy, giancarta@unica.it.

## Abbreviations

12:0 (lauric acid), 14:0 (myristic acid), 16:0, PA (palmitic acid), 16:1n7 (palmitoleic acid), 18:0 (stearic acid), 18:1n9 (oleic acid), 18:2n6 (linoleic acid), 18:3n3 (α-linolenic acid), 20:4n6 (arachidonic acid), 2-AG (2-arachidonoylglycerol), ALT (alanine transaminase), AST (aspartate aminotransferase), AT (adipose tissue), diet-C (control diet), diet-PA (PA enriched diet), EC (endocannabinoids), Elovl6 (elongase 6), FA (fatty acids), FCCP (4-(trifluoromethoxy)phenylhydrazone), HFD (high fat diets), HOMA-IR (homeostasis model assessment-estimated insulin resistance), IL-1β (Interleukin-1β), IR (insulin resistance), Lean-C (lean rats fed diet-C), Lean-PA (lean rats fed diet-PA), MCP1, (Monocyte chemoattractant protein-1), monounsaturated fatty acid (MUFA), n3HUFA (n3 Highly Unsaturated Fatty Acids), NAE (*N*-acylethanolamines), Ob-C (obese rats fed diet-C), Ob-PA (obese rats fed diet-PA), OCR (oxygen consumption rate ), OEA (*N*-oleoylethanolamine), PEA (*N*-palmitoylethanolamine), POEA (*N*-palmitoleoylethanolamine), PUFA (polyunsaturated fatty acids), respiratory control ratio (RCR), SCD, Δ-9-desaturase (stearoyl-CoA desaturase), SFA (saturated fatty acid), SOD (superoxide dismutase), TG (triglycerides).
